# Supplementary figures and images for: Identification and subcellular localization analysis of membrane protein Ycf 1 in the microsporidian Nosema bombycis
Source: PeerJ. 2022 Jul 8;10:e13530. doi: 10.7717/peerj.13530 (PMC9272817; doi:10.7717/peerj.13530)

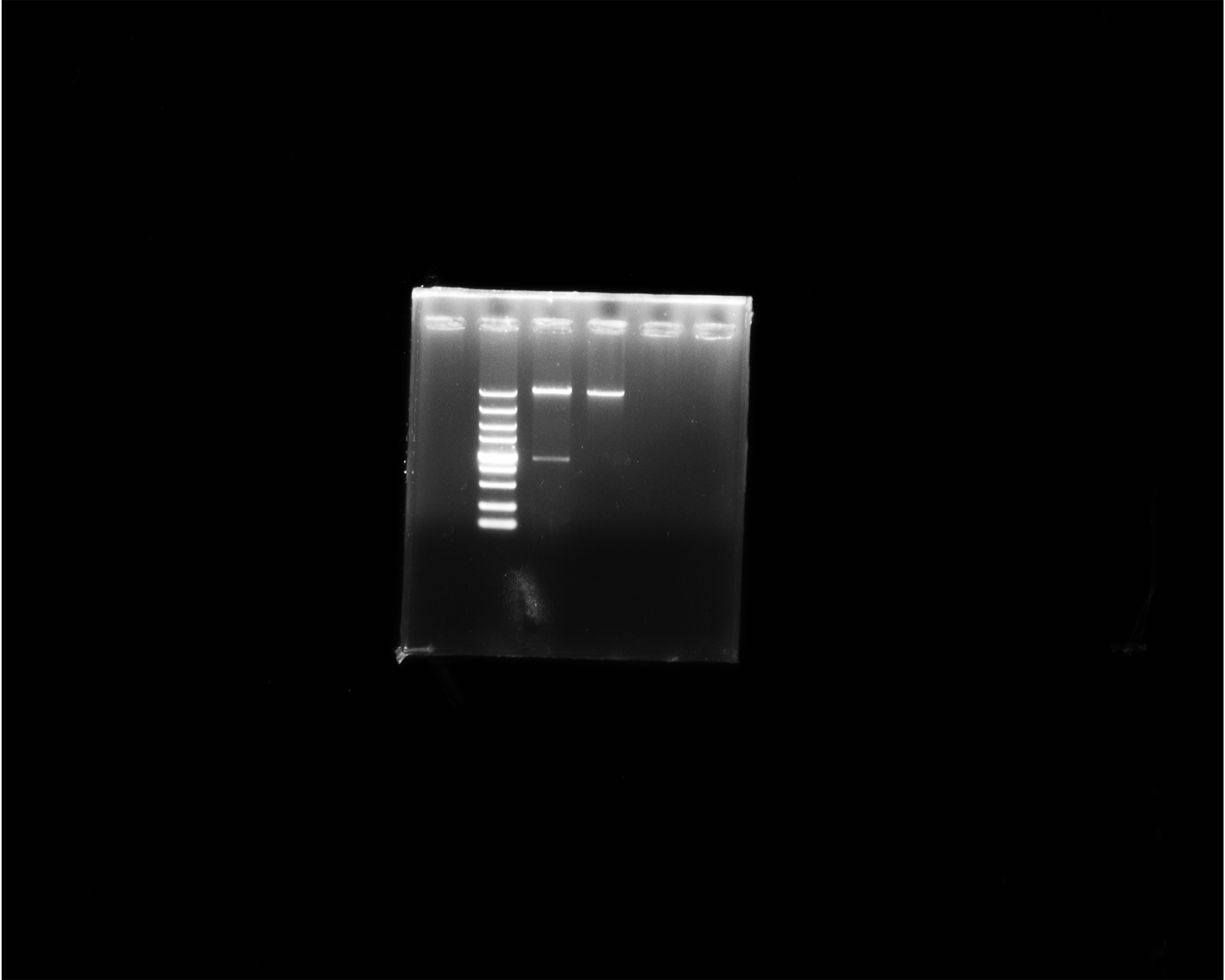

Supplement: Supplemental Information 4 [file peerj-10-13530-s004.png]

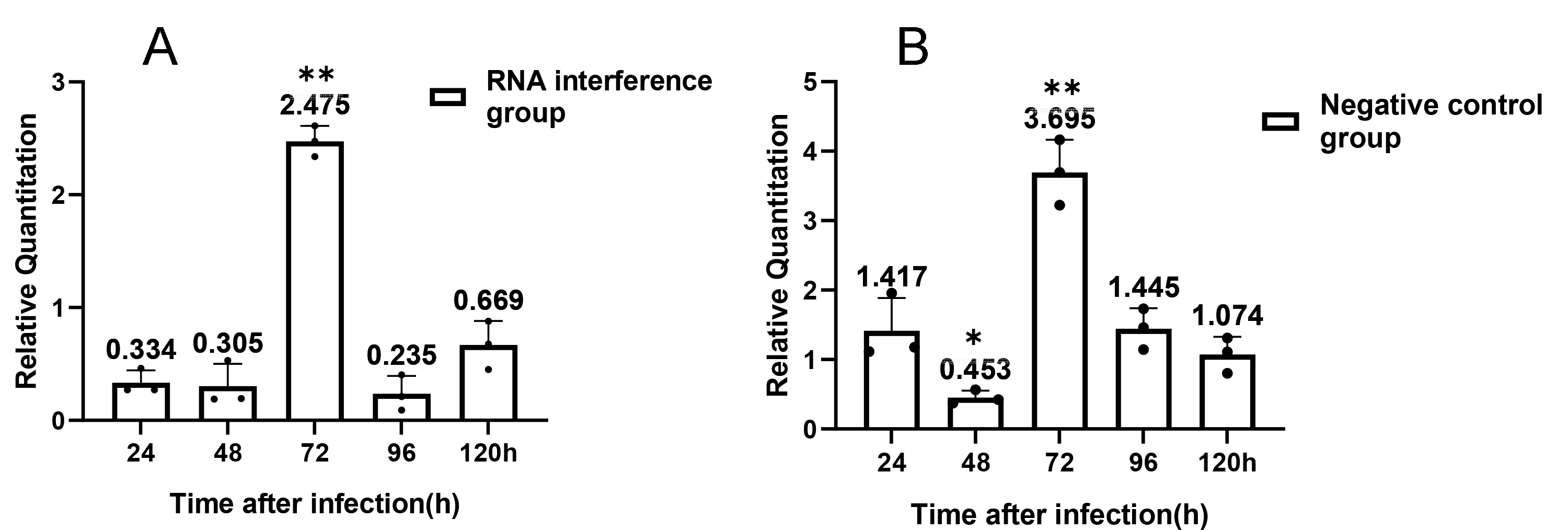

Supplement: Figure S1 — (A) relative expression of Ycf 1 gene in RNAi group. (B) relative expression of Ycf 1 gene in control group. Error bars represent the standard deviations of three independent replicates. (One-way ANOVA analysis by 24h as control. n = 3, * p < 0.05, ** p < 0.01). [file peerj-10-13530-s005.png]

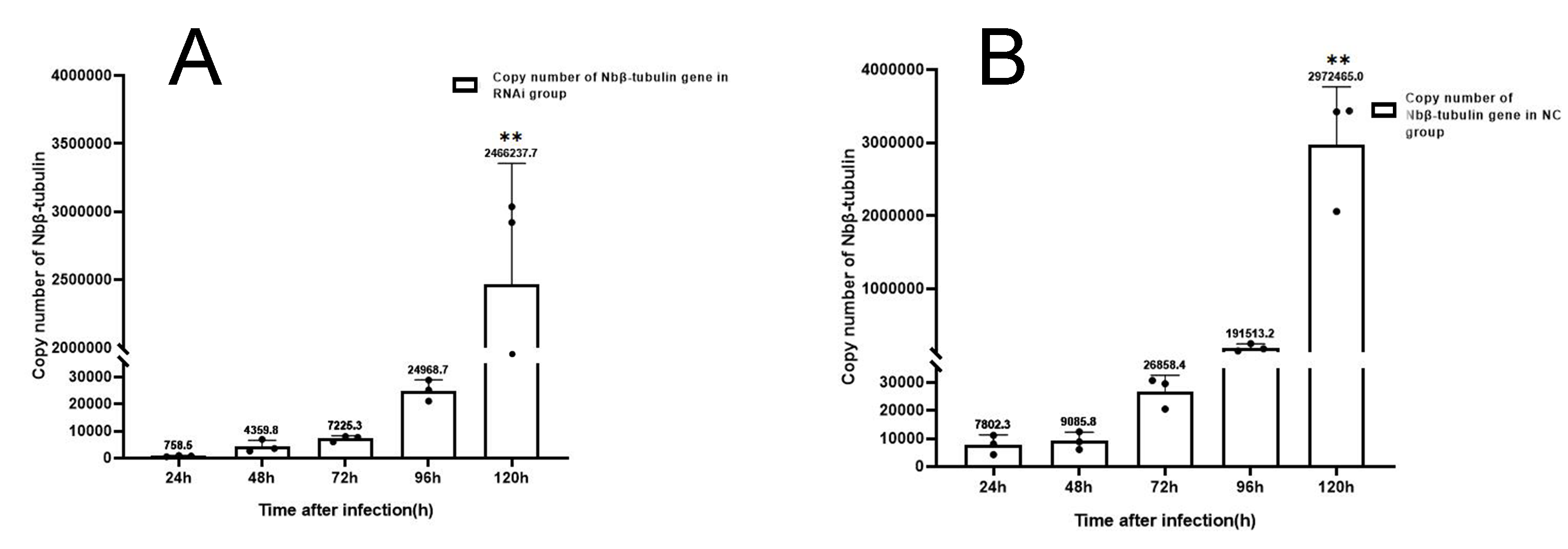

Supplement: Figure S2 — (A) Copy number of Nb β-tubulin in RNAi group. (B) Copy number of Nb β-tubulin in control group. Error bars represent the standard deviations of three independent replicates. (One-way ANOVA analysis by 24h as control. n = 3, ** p < 0.01). [file peerj-10-13530-s006.png]
