# Supplementary material for: Identification and subcellular localization analysis of membrane protein Ycf 1 in the microsporidian Nosema bombycis
Source: PeerJ. 2022 Jul 8;10:e13530. doi: 10.7717/peerj.13530 (PMC9272817; doi:10.7717/peerj.13530)
Supplement: Supplemental Information 7 [file peerj-10-13530-s007.docx]

Figure 6A Statistical Reporting

We used GraphPad Prism 8.0 (GraphPad Software, San Diego, CA, United States) to conduct the multiple t tests. Three biological replicates were set at each time point and the transcription levels were calculated by the 2^-∆∆ct^ values method. The degrees of freedom are 4, the P values of 24h, 48h, 72h, 96h and 120h are 0.017658, 0.306210, 0.01554, 0.003258 and 0.101871 respectively. Interference effect is significant at 24h, 72h, and extremely significant at 96h.

|  |
| --- |

Figure 6B Statistical Reporting

We used GraphPad Prism 8.0 (GraphPad Software, San Diego, CA, United States) to conduct the multiple t tests. Three biological replicates were set at each time point and the transcription levels were calculated by the 2^-∆∆ct^ values method. The degrees of freedom are 4, the P values of 24h, 48h, 72h, 96h and 120h are 0.024015, 0.102713, 0.003899, 0.004872 and 0.502208 respectively. The effect of inhibiting the proliferation of *N.bombycis* was significant at 24h and extremely significant at 72h,96h.

Supplementary Figure 1A Statistical Reporting

We used GraphPad Prism 8.0 (GraphPad Software, San Diego, CA, United States) to conduct the One-way ANOVA analysis by 24h as control. the P values of 48h, 72h, 96h and 120h are 0.9982, 0.0050, 0.8910, 0.2797 respectively. The effect was extremely significant at 72h.

Supplementary Figure 1B Statistical Reporting

We used GraphPad Prism 8.0 (GraphPad Software, San Diego, CA, United States) to conduct the One-way ANOVA analysis by 24h as control. the P values of 48h, 72h, 96h and 120h are 0.0218, <0.0001, 0.9999, 0.5867 respectively. The effect was significant at 48h and extremely significant at 72h.

Supplementary Figure 2A Statistical Reporting

We used GraphPad Prism 8.0 (GraphPad Software, San Diego, CA, United States) to conduct the One-way ANOVA analysis by 24h as control. the P values of 48h, 72h, 96h and 120h are >0.9999, >0.9999, 0.9999, <0.0001 respectively. The effect was extremely significant at 120h.

Supplementary Figure 2B Statistical Reporting

We used GraphPad Prism 8.0 (GraphPad Software, San Diego, CA, United States) to conduct the One-way ANOVA analysis by 24h as control. the P values of 48h, 72h, 96h and 120h are >0.9999, >0.9999, 0.9169, <0.0001 respectively. The effect was extremely significant at 120h.
