# Supplementary material for: Identification and subcellular localization analysis of membrane protein Ycf 1 in the microsporidian Nosema bombycis
Source: PeerJ. 2022 Jul 8;10:e13530. doi: 10.7717/peerj.13530 (PMC9272817; doi:10.7717/peerj.13530)
Supplement: Supplemental Information 8 [file peerj-10-13530-s008.docx]

ATGAAATTTACTACTTTGGTTTTATTTTCTATAATTACAAAAGTTTTGGCTACAACAGGGGGAGGTGATGATCCCGCTAATAAGAAAAAGGATGATTCAAAAGATAAGCAAGAAGATCCTAATAAGAAAGAACCAATGCCAGGAATAGAAGAAGGTGAATCTGAAAAAGATACTGAAAAGAAAGATACTGAACAAAAAGATACTGAAAAGAAAGATACTGGAAAGAAACCTGAAGAACCAACTACAGGTAAAGTGTCAGATGCTAACCCGCCAAAAGATCAAACCCCCGTTGGCGATATGGAAAAAAACGGCGAAGAGCTTTTAGAAGATGCCCTTAATAACGGTAAAGACAATGTTCAAAATGAAAAAAAGGATTCTAATGCTCCTGCTGATACTACTCCAGTAGTCCCCGTTGTCGATAAAGAGGCAGAGAAAAAGAAGGCTGATGAAGAAACAAAAAAGAAAGCTGAAGAAGATGCAAAAAAGAAGGCTGAAGAAGATGCAAAAAAGAAGGCTGATGAAGATGCAAAAAAGAAAGCTGAAGAAGACAAAGCAAAGCCAGCCACAGGTCCCTCTGAGACCAAGGATGAAAAATCAAGCGAAACAAAAGCAGAAACAACTTCCAGTGAT

AAACCAGCTTCAAGTGATGTCCTTAAGAAAGACACTGATTCATCTGATCCTAAACCACCCAGCTCAACTGATAAAAACACTGCTGAACCTTCTACTACAGAAAAAGCCCCTGCTCCAGGAGCCAATCCTCCTGCTGATGGAGAGCCTAAAAAAGATGGAGCCGGCCCTGCTCCAGGATCAGGACCCGAACCTCCTGTTAAAGACCTGGCCAAGAATGATACAACTGACGTTAAAAAGAATGAAAAGTCTTCGAATTGGAAATCATATTTACTCTGGGGAGCACTCGGTCTTTGTGTAGTCGGATTCGTTGTTGTTGTGGCTATGATGGCTTCTAAATAA
